# A Novel Surgical Technique for the Management of Large-Volume Neurogenic Heterotopic Ossification Following a Spinal Cord Injury: The Sashimi Technique

**DOI:** 10.7759/cureus.60966

**Published:** 2024-05-23

**Authors:** Akira Toga, Ayush Balaji, Toshiyuki Yamauchi, Atsushi Funayama

**Affiliations:** 1 Department of Orthopaedic Surgery, Saiseikai Yokohamashi Tobu Hospital, Yokohama, JPN; 2 Department of Medical Education, Hull York Medical School, York, GBR

**Keywords:** functional outcomes, hip, surgical resection, spinal cord injury, heterotopic ossification

## Abstract

This case series investigates the efficacy of the "sashimi technique," a novel surgical approach utilizing a curved chisel for the resection of heterotopic ossification (HO). The main focus is on reducing resection margins and preventing excessive bone removal while maintaining optimal functional outcomes and preventing recurrence. Two cases illustrate successful outcomes in patients with spinal cord injuries and severe HO of the hip, emphasizing the precision of using the curved chisel-based technique in improving patient mobility while still achieving a desired resection margin. The study highlights the effectiveness of using a curved chisel in protecting neurovascular structures and maintaining resection precision. Additionally, the integration of postoperative radiotherapy and pharmacological treatment is emphasized as a strategy to prevent recurrence. The goal of this procedure is to improve functional outcomes and patient quality of life.

## Introduction

Heterotopic ossification (HO) is a pathological condition characterized by abnormal bone formation in soft tissues, often observed following central nervous system injuries. This case series examines the incidence, management, and outcomes of HO in patients. It underscores the complexity of diagnosing and treating HO, highlighting the need for a multidisciplinary approach. The series also explores innovative surgical techniques and postoperative care strategies aimed at reducing recurrence and improving patient mobility. This case series focuses on a novel surgical technique to manage HO developed to enhance patient outcomes by reducing resection of areas of unaffected bone during surgical treatment. In HO, aberrant bone formation in soft tissues significantly impairs mobility and quality of life post-injury. Our approach integrates advanced surgical methods with comprehensive postoperative care, addressing the unique challenges of HO.

## Case presentation

Case 1

**Figure 1:**
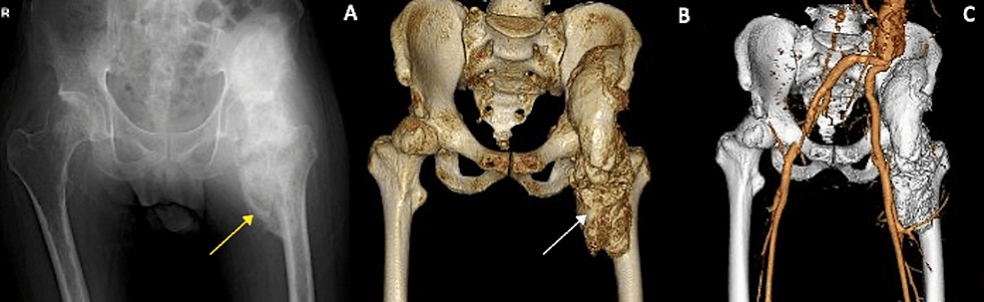
Patient 1 preoperative images Patient 1 preoperative X-ray (A), CT scan of the hip (B), and CT angiogram reconstruction of the vasculature (C). The yellow arrow depicts heterotopic ossification visible on X-ray. The white arrow shows the extent of heterotopic ossification on the CT scan. CT: computed tomography

The first case was a 33-year-old male with a history of spinal cord injury, suffering from severe HO of the hip. He had a history of paraplegia due to spinal cord infarction (Th8 AIS A) one year prior. This was after surgery for a type A aortic dissection thoracoabdominal aortic aneurysm due to residual dissection. At age 28, he experienced an initial type A dissection, which was repaired with an artificial graft and a stent graft. The patient had restricted mobility due to extensive hip joint ossification, making it difficult to maintain a seated posture. The ossification is seen visibly on the preoperative X-ray and shows space restriction (Figure 1).

**Figure 2:**
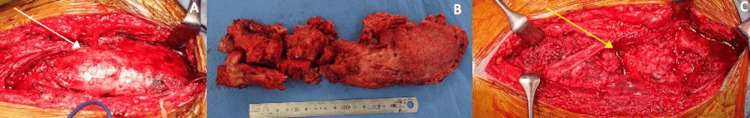
Patient 1 intraoperative images Pre-resection (A), resected ossification (B), and post-resection image of the femur (C). The white arrow shows smooth heterotopic ossification surrounding the femur. The yellow arrow shows the post-resection state of the femur.

The patient also had a computed tomography (CT) scan and CT angiography to map the path of the vasculature surrounding the femur and heterotopic ossification (Figure 1). The "sashimi technique" was employed for precise ossification removal in this patient through the Smith-Peterson approach. Ectopic bone (300 g) was removed from the site using osteotome and bone saw initially, and then, a curved chisel was used to resect the bone close to the native femur (Figure 2).

**Figure 3:**
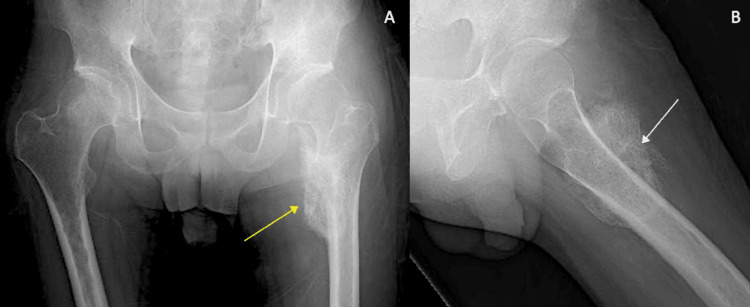
Patient 1 postoperative X-ray Anteroposterior (A) and lateral view (B). The yellow arrow shows the remaining heterotopic ossification post-resection. The white arrow shows the remaining heterotopic ossification post-resection on the surface of the femur.

The curved chisel was also used in areas in close proximity to any neurovascular structures. Postoperative X-ray showed good resection margins (Figure 3).

Postoperatively, the patient had improved hip flexion from 20° to 80°. Operative bleeding was 1,297 mL. The postoperative treatment included radiation therapy two days postoperatively to prevent recurrence (8 Gy × 1 time), and the patient was also given oral etidronate disodium 800 mg. The patient was discharged from the hospital six days after surgery. At 10-month postoperative follow-up, there was no recurrence or signs of ossification. The patient reported that this significantly enhanced sitting comfort and daily activities, allowing the patient to be more mobile.

Case 2

**Figure 4:**
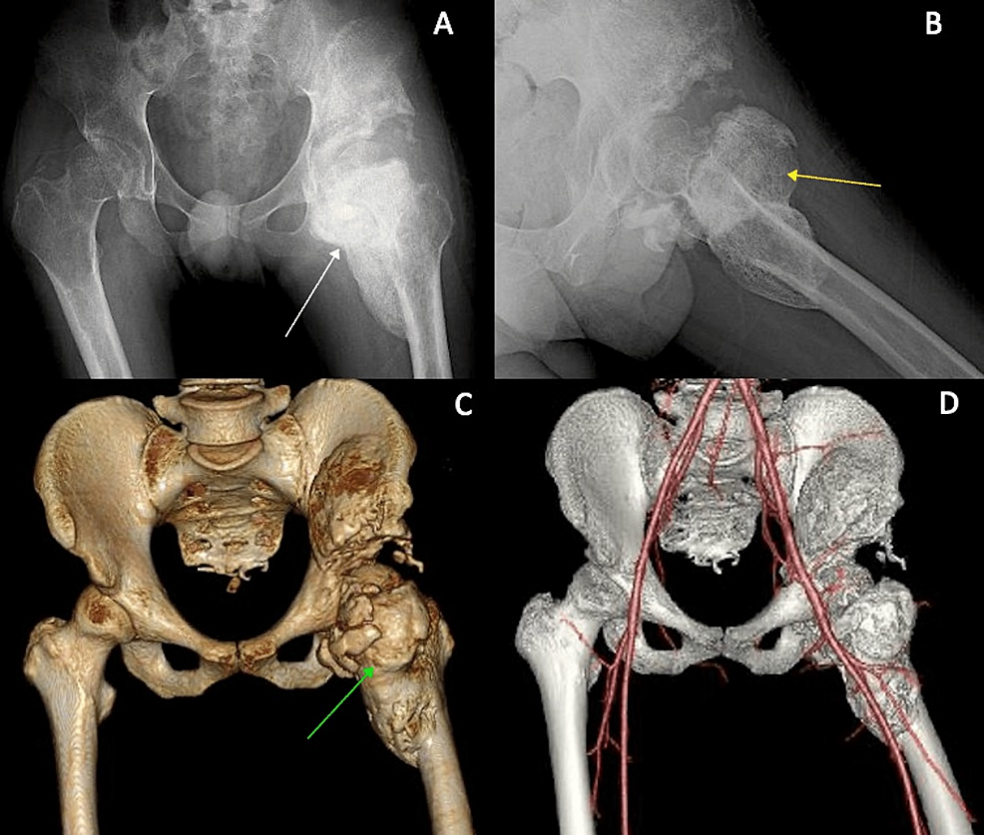
Patient 2 preoperative images Preoperative anteroposterior (A) and lateral X-ray (B), CT scan of the hip (C), and CT angiogram reconstruction of the vasculature (D). The white arrow depicts the wide extent of heterotopic ossification of the femur toward the medial aspect of the femur. The yellow arrow depicts the wide extent of heterotopic ossification of the femur. The green arrow depicts the wide extent of heterotopic ossification of the femur and erosion of the pelvis on CT scan. CT: computed tomography

The second case involved a 23-year-old male with a cervical spinal cord injury (C6 AIS A) who experienced recurrent heterotopic ossification (HO) of the hip. He presented with persistent pain and limited range of motion (ROM) in his left hip. The injury occurred one year earlier during a company trip when he jumped into a pool, resulting in the need for C4-C6 posterior decompression and fixation surgery. Partial resection of the ectopic ossification in the left hip was performed at another hospital one year after his posterior decompression and fixation surgery. However, 10 months after this resection, he continued to have a limited range of motion, with severe restriction in hip flexion and adduction. He requested a second opinion and was referred to our hospital. The remaining ossification is seen visibly on the preoperative X-ray and also showed a significant reduction in the joint space (Figure 4).

**Figure 5:**
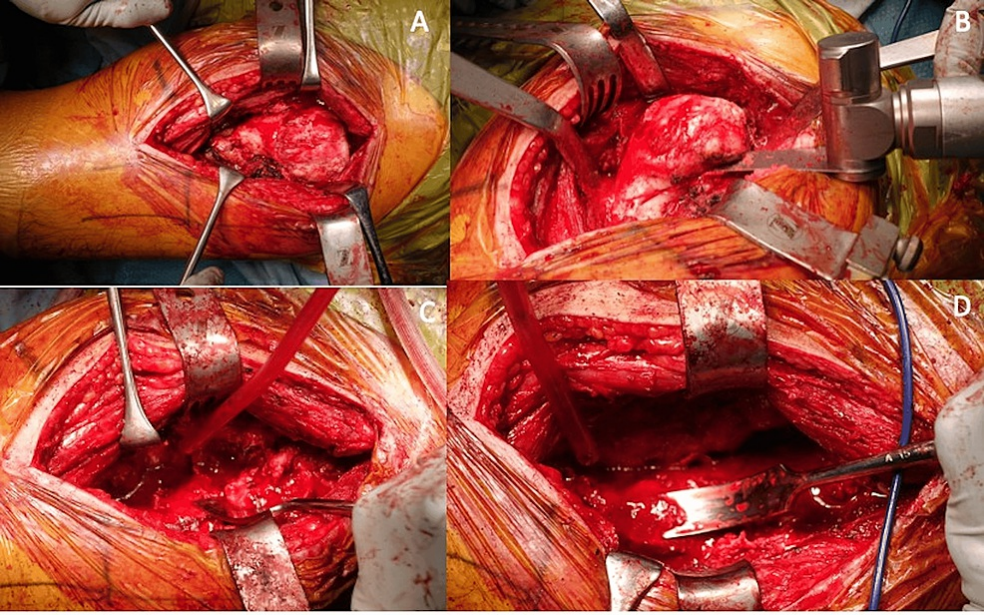
Patient 2 intraoperative images Pre-resection view of the femur with heterotopic ossification exposed from the surrounding tissue (A). Utilization of bone saw for gross resection of ossification at a safe level, avoiding neurovascular structures and excessive removal of healthy bone (B). Fine resection using a curved chisel to precisely remove the remaining ossified tissue while preserving native anatomy (C and D).

The patient also had a CT scan and CT angiography to map the path of all vasculature in close proximity to the femur and heterotopic ossification (Figure 4). A resection was planned using the sashimi technique, and a total of 212 g of ectopic ossification bone was removed (Figure 5).

**Figure 6:**
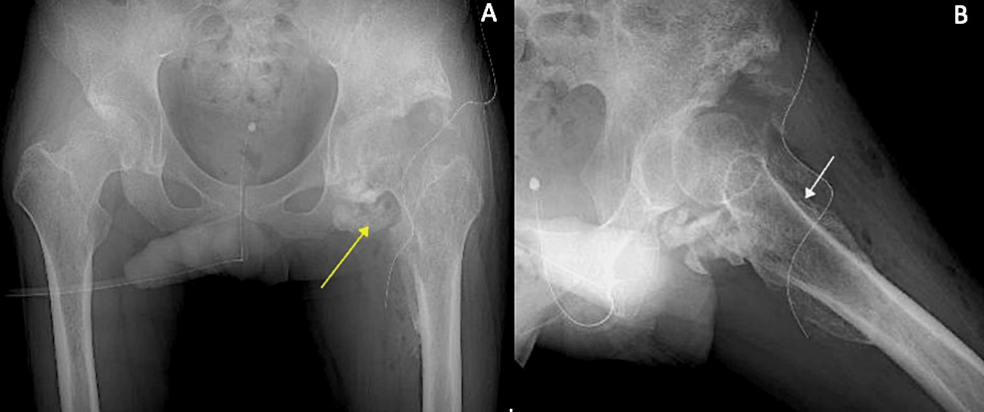
Patient 2 postoperative X-ray of the hip joint Anteroposterior (A) and lateral view (B). The yellow arrow shows the post-resection remainder of the heterotopic ossification. The white arrow depicts the extent of resection on the femoral surface in the lateral view.

Postoperative X-ray showed good resection margins (Figure 6).

His hip flexion increased from 50° to 80°, adduction increased from -10° to 20°, and abduction increased to 40° from 35°. Operative bleeding was 627 mL. The postoperative treatment included radiation therapy two days postoperatively to prevent recurrence (8 Gy × 1 time), and the patient was also given oral etidronate disodium 800 mg. During follow-up, the patient mentioned increased joint mobility, aiding in smoother wheelchair transfers and daily functions. There was no recurrence reported six months after surgery.

In both cases, joint mobilization training was started one week after surgery. The curved chisels used in our cases were of set specifications (radius, 40 or 65 mm; length, 48.5 or 65 mm; width, 15 or 20 mm) (Mizuho, Tokyo, Japan).

## Discussion

**Figure 7:**
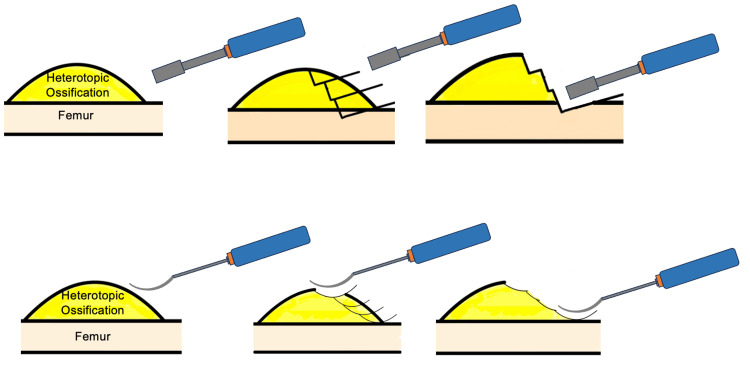
Technique of resection using the sashimi technique compared to conventional resection technique This figure describes the difference in resection strategies between the conventional resection method (depicted in the upper section of the image) and the sashimi technique (depicted in the lower section of the image). The sashimi technique allows for the preservation of more native bone as compared to the conventional resection method. The figures were constructed by the authors.

These cases underscore the effectiveness of our developed "sashimi technique" in managing HO. The nomenclature behind the name "sashimi technique" is inspired by the preparation of "sashimi" in Japanese cuisine, where a chef uses a knife to very carefully cut the fish into thin slices. Just as a skilled sushi chef meticulously slices fish with a sharp, curved blade to maintain the integrity and texture of each piece, our surgical technique utilizes a curved chisel to carefully resect the heterotopic ossification, similar to a piecemeal resection approach, while preserving the surrounding healthy bone and soft tissue. This approach, characterized by precise and minimal removal of unaffected bone, aims to enhance postoperative range of motion (ROM) and alleviate patient symptoms. In conventional resection, a straight, flat-edged chisel is used to resect the ossified tissue in perpendicular angles, and approaching the edge of healthy bone, the angulation is changed to reduce the volume of tissue resected; however, this does not always preserve all the healthy tissue (Figure 7).

**Figure 8:**
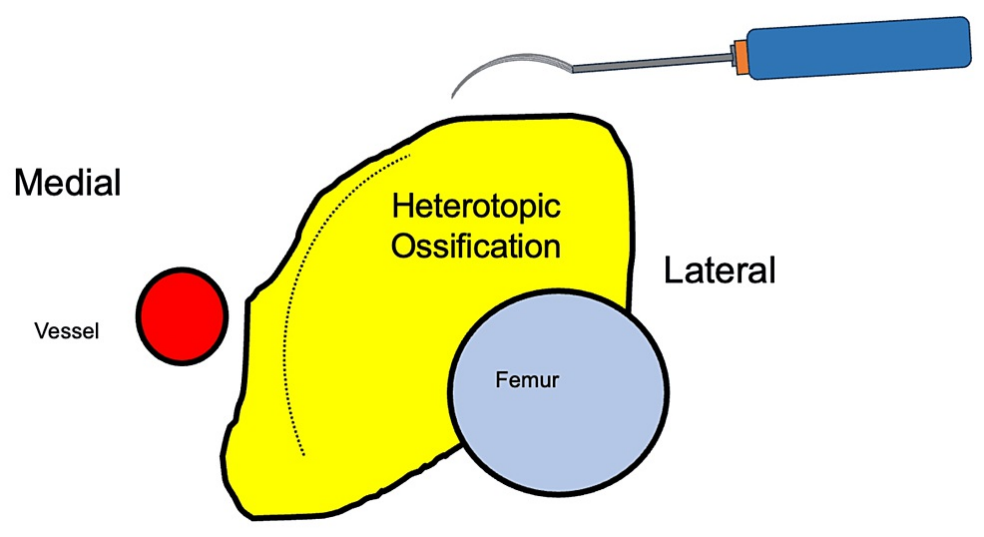
Demonstration of sashimi technique and use case demonstrating margins This figure depicts an axial view of the sashimi technique and shows the curved path of the resection. The curvature of the chisel also allows for the protection of the vessel in close proximity. The figure was constructed by the authors.

In our technique, a curved chisel is used to allow for better maintenance of margins during resection (Figure 8).

This surgical technique incorporates the principles of high precision, a minimally invasive approach, and a comprehensive understanding of both anatomy and pathology during HO resection. Intraoperative challenges include the critical task of distinguishing between normal bone and ectopic ossification. Equally important is the preservation of vital vascular structures, such as the femoral artery, to ensure sufficient blood flow to the femoral head and minimize the risk of ischemic complications. The utilization of 3D models in preoperative planning emerges as a crucial tool for understanding the complex anatomy and extent of HO. The potential risks associated with HO resection include intraoperative fractures and perioperative complications. Emphasis on surgical interventions within reasonable limits echoes the sentiment of minimizing unnecessary risks. Recent research sheds light on the impact of hip HO on function and potential management strategies [1]. Studies highlight significant pain reduction and improved function after complete surgical resection. However, when resection margins include a significant amount of native bone, issues such as bleeding, stability, fracture, and functional loss can be a concern [2,3]. Minimally invasive techniques such as computer navigation show promise for improved outcomes [4]. Reduced tissue disruption is closely associated with faster healing processes, diminished postoperative pain, and a lowered incidence of complications, enhancing the overall recovery experience for patients. Moreover, the preservation of healthy tissue around the site of HO is instrumental in ensuring better long-term outcomes in terms of joint mobility and function, directly contributing to improved patient quality of life. Additionally, conserving surrounding tissue not only facilitates immediate recovery but also preserves options for future surgical interventions or treatments, offering a strategic advantage in long-term patient management. To improve the safety of the procedure, it is important to use intraoperative C-arm X-ray guidance to visualize both the resection margins and understand the anatomy during resection. Continuous range of motion assessment intraoperatively is also crucial to ensure good functional outcomes postoperatively. It is important to take care and avoid resecting into the joint, the articulating surfaces of the femur with the pelvis, as this can cause instability and increase the risk of intraoperative fracture.

The need for thorough patient education to manage expectations and inform them about potential complications is also underscored. The concept of preoperative embolization is introduced as a strategy to facilitate HO removal, with a need for joint input and consultation from vascular surgery. The potential benefits of embolization, such as simplifying the identification and dissection of blood vessels are a key factor; however, there is a need for careful consideration and weighing the risks [5,6]. In these cases, we opted not to perform embolization to preserve blood flow to the bone. This was primarily to avoid avascular necrosis of the femoral head and understand that this choice could increase intraoperative bleeding and present additional challenges during surgery.

The advantages of the instrument used, the curved chisel, in the sashimi technique include how it allows for finer movements and more targeted tissue removal. The curved chisel enhances surgical precision while minimizing the risk of inadvertent damage to surrounding structures. Moreover, the curvature of the chisel facilitates better force distribution, reducing trauma and enhancing overall surgical outcomes. Previous reports of similar uses are seen in the literature for a use case in osteosarcoma resection of the femur [7]. Another case was reported in the literature regarding sarcoma resection in the sternum in crucial areas [8].

It is pertinent to highlight the importance of preoperative planning in optimizing the use of the curved chisel technique for resection so as to not compromise resection margins. It has been suggested in the literature that imaging modalities such as magnetic resonance imaging (MRI) can be used to assess the resection margins postoperatively [9]. The sashimi technique, combined with postoperative radiotherapy and etidronate disodium, aims to reduce the recurrence of heterotopic ossification (HO). Although our case series of two patients is limited and does not provide definitive evidence, existing literature suggests that pharmacological treatments and radiotherapy can effectively reduce recurrence rates [10,11]. It is crucial that postoperative management is optimized to prevent recurrence and further range-limiting masses. Different strategies, including combination therapy with radiotherapy and nonsteroidal anti-inflammatory drugs (NSAIDs), have proven effective [10,11]. Further research is needed to confirm the effectiveness of combining surgical resection with these treatments. Bisphosphonate such as ethindronate was also shown in the literature to be the best choice for pharmacological treatment of already established HO, thus making it our agent of choice [12]. It is suggested in the literature that serum alkaline phosphatase (SAP) can be used as a marker for heterotopic ossification. Therefore, it might be utilized as a marker for predicting recurrence through baseline monitoring of SAP immediately postoperatively and then measured during follow-up [13]. The timing of resection is still debated; however, it seems to be the general consensus that earlier resection is better to reduce pain and prevent deterioration of function [14]. Further considerations include the choice of imaging modality in the preoperative planning process. A combination of CT with orthogonal X-ray projections may help improve the quantification of the ossification [15]. In our patients, it is key that more than the resection margins, the range of motion was prioritized to ensure good functional outcomes. It is key to evaluate the functional needs of the patient prior to determining the operative course.

## Conclusions

The presented case serves as a platform for ongoing learning and improvement in surgical techniques. The participants express optimism for future cases and the continued accumulation of knowledge to refine and optimize the approach to HO resection, ultimately improving patient outcomes in this challenging clinical scenario. Combining detailed preoperative planning with precise intraoperative techniques offers a promising path to improving surgical outcomes, reducing recurrence risks, and enhancing the overall quality of life for patients with HO. It is key to explore the patient's functional status, including but not limited to an assessment of the patient's preoperative mobility status, and frailty, as well as a multifactorial assessment of the patient's ability to perform activities of daily living. Following this, it is important to address their expectations to determine the best course of action.
